# Sewing Needle Penetration into Thorax: What Might be the Cause? 

**DOI:** 10.21699/ajcr.v8i2.560

**Published:** 2017-03-18

**Authors:** JD Rawat, Sudhir Singh, Digamber Chaubey, Gurmeet Singh

**Affiliations:** Department of Pediatric Surgery, KGMU, Lucknow, India

**Dear Sir**

A 10-month-old male baby presented with irritability and inconsolable cry for last two weeks which increased on touching the chest. There was no history of fever, respiratory symptoms, or any accident. On examination, an indurated tender nodule over right lower chest was present. Chest x-rays showed a metallic foreign body at right lower chest extending from skin to pleural cavity (Fig.1A,1B). Foreign body was removed under anesthesia. It was a two inches long sewing needle. Child was discharged on day three of hospitalization after information to authority for further action.

**Figure:**
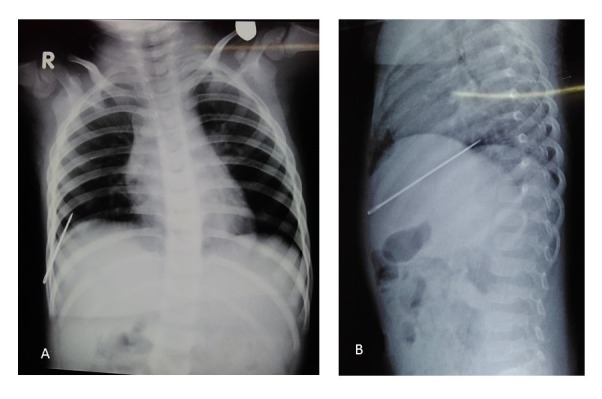
Figure 1: Chest radiograph A- PA and B-Right lateral view showing metallic foreign body in right lower chest going towards right pleural cavity.

Several reports of insertion of needles into various body parts of children have appeared in the medical literature.[1] These cases generally have other findings of abuse, such as bruises, abrasions, or burns on other part of body.[2] As there was no history of accidental fall leading to accidental insertion of needle, a deliberate insertion by someone cannot be ruled out and should raise a strong suspicion of child abuse.

## Footnotes

**Source of Support:** Nil

**Conflict of Interest:** None declared

